# Surgical Management of an Impacted Mandibular Second Premolar in Close Proximity to the Mental Foramen: A Case Report

**DOI:** 10.3390/reports8030177

**Published:** 2025-09-15

**Authors:** Aikaterini Blouchou, Panagiotis Rafail Peitsinis, Ioannis H. Makrygiannis, Gregory Venetis, Ioannis Tilaveridis

**Affiliations:** 1 School of Dentistry, Aristotle University of Thessaloniki, 54124 Thessaloniki, Greece; ppeitsini@dent.auth.gr; 2 Department Oral and Maxillofacial Surgery, Aristotle University of Thessaloniki, 54124 Thessaloniki, Greece; makjohn-dent@live.com (I.H.M.); grvenetis@gmail.com (G.V.); jtilaver@yahoo.com (I.T.)

**Keywords:** tooth impaction, mental foramen, mandibular second premolar, cone-beam computed tomography (CBCT), surgical extraction, platelet-rich fibrin (PRF)

## Abstract

**Background and Clinical Significance**: Tooth impaction is a developmental anomaly characterized by the inability of a tooth to emerge into its predetermined anatomical position within the oral cavity during the normal eruption period. Impaction of the mandibular second premolar is an uncommon condition and poses a heightened risk of neurosensory injury when the tooth is adjacent to the mental foramen. Early diagnosis and precise planning are therefore essential. **Case Presentation**: This case report presents a rare instance of an asymptomatic impacted mandibular second premolar located in close proximity to the mental foramen in a 44-year-old female patient. The impaction was discovered incidentally on an orthopantomogram, and Cone-Beam Computed Tomography (CBCT) confirmed intimate contact between the root of the impacted second premolar and the mental nerve. Surgical removal was performed under local anesthesia via a conservative triangular flap and a corticotomy window. Platelet-Rich Fibrin (PRF) generated from autologous blood was placed in the socket to foster healing. The proximity of the mental foramen dictated minimal bone removal and atraumatic luxation to avoid nerve stretch or compression. PRF was selected as an effective biomaterial shown to accelerate soft tissue healing and moderate postoperative discomfort, potentially reducing the likelihood of neurosensory disturbance. The socket presented satisfactory healing, and neurosensory function was normal at the first week follow-up and remained normal at 7 months postoperatively (longest follow-up), and no complications were reported by the patient. **Conclusions**: CBCT-guided planning, meticulous surgical techniques, and adjunctive PRF allowed for safe extraction without post-operative paraesthesia. Timely identification of such rare impactions broadens treatment options and minimizes complications.

## 1. Introduction and Clinical Significance

The development of human teeth involves complex molecular interactions and tissue changes between the oral ectoderm, responsible for enamel formation, and the mesenchymal tissues that form other tooth structures [1,2]. Eruption is a physiological process whereby a tooth emerges through the alveolar bone into the oral cavity [3]. Typically, eruption occurs when the root length reaches two-thirds to three-quarters of its final length [4]. Several factors influence eruption, including genetics, gender, nutrition, body height and weight, preterm birth, craniofacial morphology, hormonal influences, systemic diseases, and syndromes such as cleidocranial dysostosis and hypothyroidism [5,6]. These factors can disrupt normal eruption timing, leading to impaction.

Impaction is defined as the failure of a tooth to erupt into its expected position within the stipulated timeline [7]. Common causes include ectopic eruption pathways, loss of space in the dental arch, ankylosis of deciduous teeth, and supernumerary teeth [6]. Demographic studies reported an estimated prevalence that ranges from 0.8% to 3.6%, while the preponderance of cases is associated with third molars [8]. Following wisdom teeth, impacted maxillary canines and mandibular second premolars are most common, with lower prevalence rates seen in incisors, mandibular canines, and molars [9]. In the adult population, mandibular second premolars account for 0.2–0.3% of the total impaction prevalence, with a typical eruption age between 11 and 12 years; however, this can vary due to multiple factors [10,11,12].

Despite the fact that impacted teeth generally remain asymptomatic, they can potentially lead to caries, pericoronitis, cystic lesions, tumor formation or even resorption of neighboring roots [13]. When impacted teeth or associated cysts are near neural structures like the mental or inferior alveolar nerves, complications such as paresthesia can occur [14]. Dental practitioners often detect impacted teeth incidentally on radiographs, emphasizing the importance of early diagnosis. In cases where surgical removal is imperative, specialized oral and maxillofacial surgeons are equipped to perform the procedure while safeguarding critical anatomical structures such as the inferior alveolar nerve, mental nerve, maxillary sinus, lingual nerve, and vital blood vessels [15]. Proper planning reduces the risk of intraoperative complications and guarantees successful surgical management.

A notable evolution in biomedical sciences was the introduction of Platelet-Rich Fibrin (PRF), a second-generation platelet concentrate and advancement over Platelet-Rich Plasma (PRP) [16]. This biomaterial, produced by the patient’s blood by centrifugation, constitutes a resorbable fibrin matrix, rich in platelets, cytokines, cells and growth factors. These molecules are released in the surrounding tissues, enhancing healing and tissue regeneration, including promotion of bone tissue repair [17,18]. Nowadays, PRF is now widely used in many aspects of dentistry such as oral and maxillofacial surgery, periodontology and implant dentistry [16]. Even in complex extraction cases of impacted teeth, PRF is gaining ground among other emerging factors that can promote wound healing including collagen sponges, graft materials, hydrogels, nanofiber scaffolds [19].

This case report describes the surgical extraction of the left mandibular second premolar in close proximity to the mental nerve followed by PRF application. The referred case is noteworthy for combining the rare prevalence of the mandibular second premolar impaction, the diagnostic accuracy of Cone-Beam Computed Tomography (CBCT) indicating the proximity to the mental nerve and the advantages of PRF to optimize wound healing with the aim of minimizing morbidity and reducing pain and swelling.

## 2. Case Presentation

### 2.1. Initial Visit, Radiographic Evaluation and Diagnosis

A 44-year-old female patient was referred to the Department of Oral and Maxillofacial Surgery at the Dental School of Aristotle University of Thessaloniki for the surgical extraction of an impacted mandibular second premolar, specifically tooth number #35. The patient was informed of all the available treatment options, including the advantages and the contingent risks of each alternative. After thorough assessment of the suggested treatment plans, the patient chose the surgical extraction of the impacted tooth followed by an implant placement and a prosthodontic restoration, in order to subsequently rehabilitate the edentulous area. Informed consent was acquired from the patient and CARE guidelines were followed. Tooth numbering followed the World Dental Federation (FDI) system.

**Figure 1:**
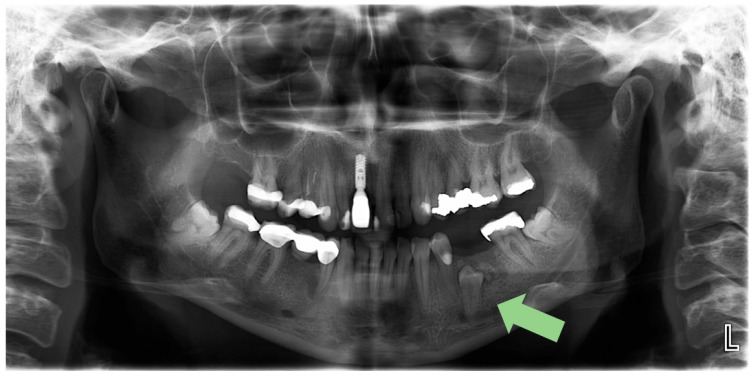
Orthopantomogram (OPG) of the patient and the arrow indicates the impacted mandibular left second premolar.

**Figure 2:**
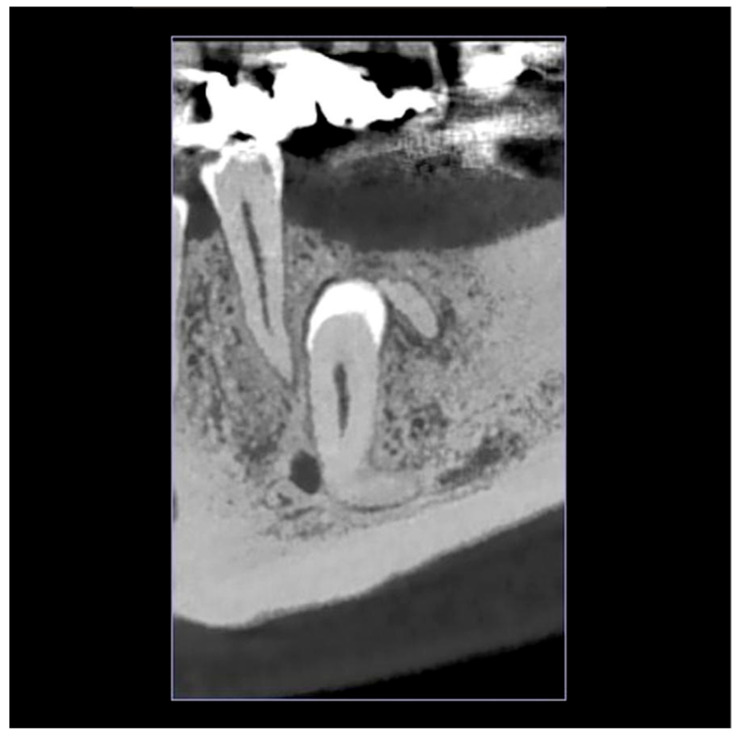
CBCT view showing the impacted second premolar (#35) in proximity to the mental foramen.

The impacted left mandibular second premolar was initially identified incidentally on an orthopantomogram (OPG) taken on 31 May 2024 [Figure 1]. A subsequent Cone-Beam Computed Tomography (CBCT) scan on 14 June 2024 was performed to precisely locate the tooth and assess its relationship to the mental foramen, confirming the complete bony impaction of tooth #35 and the proximity to the mental nerve [Figure 2].

The patient’s medical history was unremarkable, with no systemic conditions or medications. Dental history revealed a fistula associated with tooth #44. Clinical examination of the left side of the mandible showed no signs of inflammation, and the patient reported no pain or tenderness. Radiographs indicated no cystic pathology related to the impacted tooth #35, although the crown appeared to contact the residual mesial root of #36. CBCT imaging showed the root of tooth #35 to be adjacent to the mental nerve, forming an angle of roughly 90° to the occlusal plane.

### 2.2. Surgical Intervention

**Figure 3:**
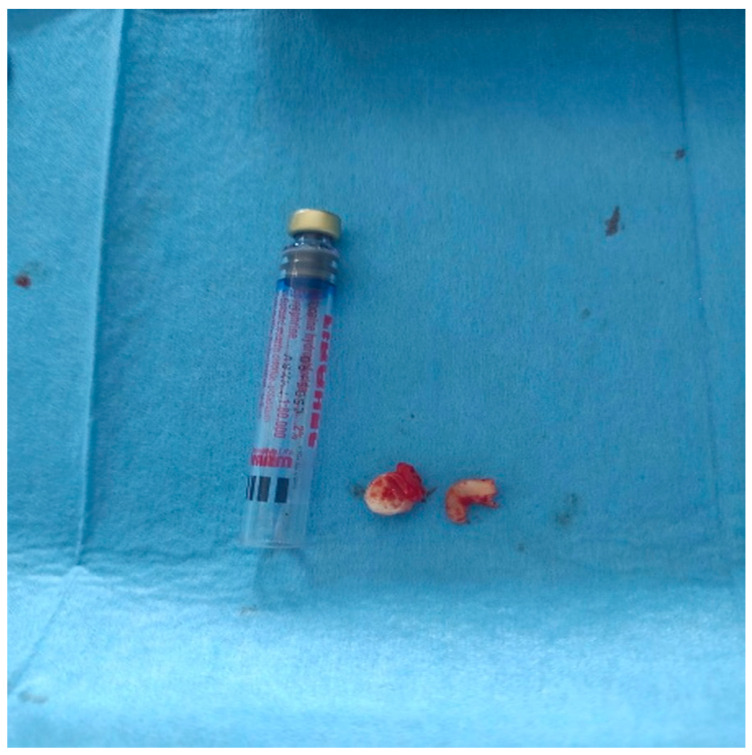
The extracted second premolar.

Based on clinical and radiographic findings, the treatment plan involved surgical extraction of the residual mesial root of #36 along with the impacted tooth #35, supplemented by PRF application to promote healing [Figure 3]. Before the surgical intervention, blood was drawn via venipuncture from the median cubital vein into a sterile, anticoagulant-free vacutainer tube, which was centrifuged at 2700 rpm for 12 min to produce L-PRF [20]. Afterwards, L-PRF was subsequently applied to the surgical site.

Local anesthesia was administered via left inferior alveolar nerve block and mental nerve block using 3% Mepivacaine. A full-thickness mucoperiosteal triangular flap was raised through a crestal incision on the edentulous alveolar ridge extending from the mesial aspect of #37 to the buccal aspect of #34, with additional vertical releasing incisions.

**Figure 4:**
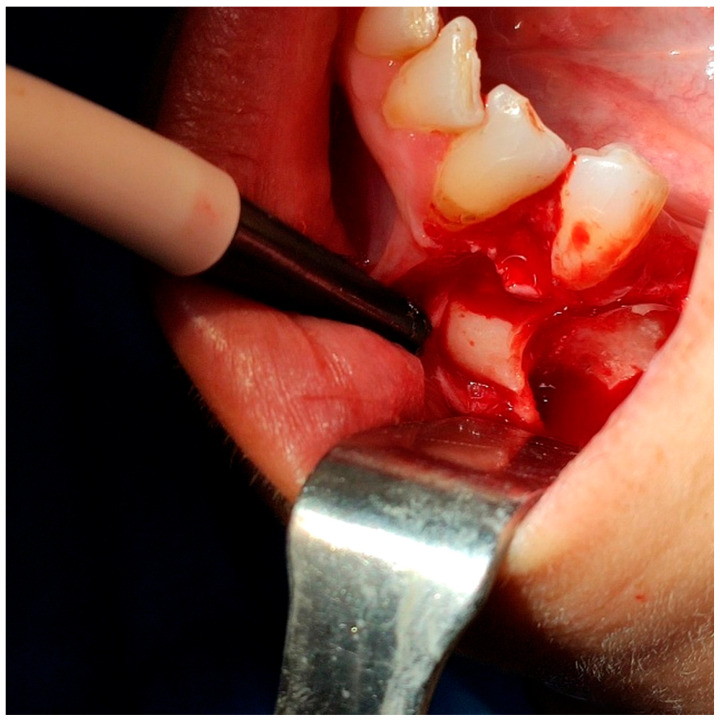
The surgical field after the extraction.

**Figure 5:**
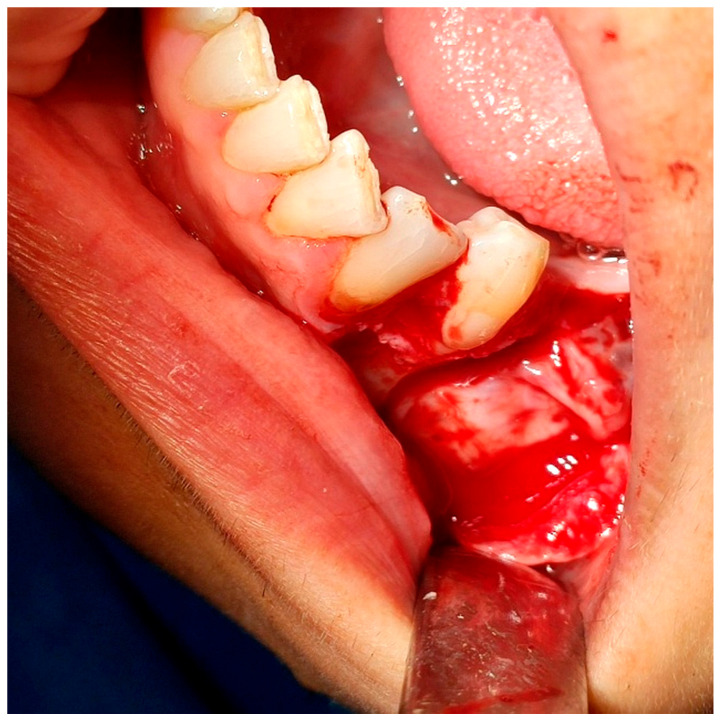
The PRF membrane covers the defect.

Based on the CBCT data, a bony window was created using a rotary bur under continuous saline irrigation to expose and luxate the tooth. An additional odontotomy was performed with a round bur under irrigation to separate the crown from the root. As a result, luxation of each part was more effortless, avoiding further apical pressure toward the mental foramen and the tooth was removed in fragments. After the extraction, the socket was thoroughly irrigated with normal saline to remove debris [Figure 4]. The PRF clot was placed within the socket and covered with a PRF membrane, stabilized using interrupted 3-0 silk sutures. Soft tissues were repositioned and sutured to promote healing [Figure 5].

### 2.3. Postoperative Management

**Figure 6:**
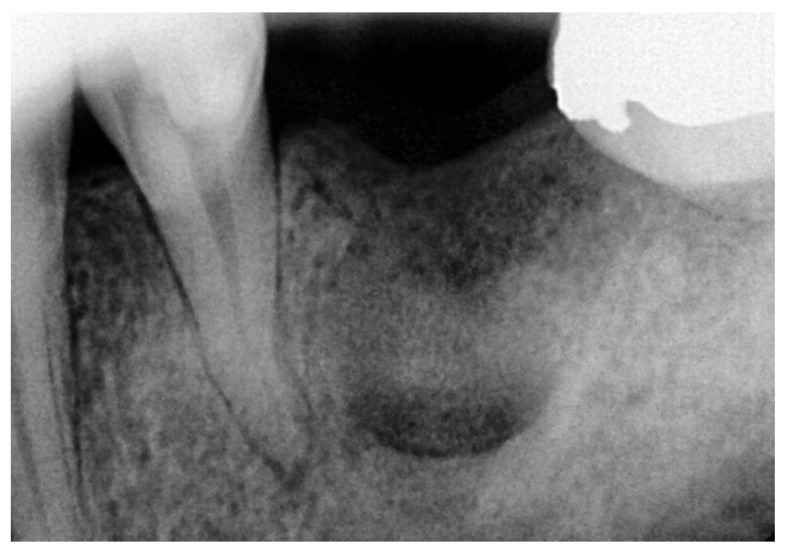
Postoperative periapical radiograph after 7 months (longest follow-up).

Postoperative instructions were provided. The patient received antibiotics per os (amoxicillin with clavulanic acid 500/125 mg) three times daily for five days; corticosteroids (methylprednisolone 4 mg) two times daily for two days; and then a reduced dosage of methylprednisolone to 2 mg, instructing the patient to take the medication per os two times daily for one day. No complications were observed post-surgery. During the one-week follow-up on 4 July 2024, the patient reported normal sensation. The sutures were removed and the surgical site presented satisfactory healing. On 27 January 2025, the patient attended the second follow-up visit (longest follow-up), at 7 months postoperatively. Clinical examination showed normal neurosensory function, with no complications reported during this period. Additionally, a periapical radiograph was taken to validate partial bone healing in the extraction socket [Figure 6].

## 3. Discussion

Impaction constitutes a moderately common developmental condition in the craniofacial region, occurring in approximately 0.8–3.6% of the population [8]. Conversely, impaction of mandibular second premolars is relatively rare, with literature estimating prevalence at about 0.2–0.3%, often posing a risk due to proximity to the mental nerve, especially depending on impaction depth and type [21].

Impacted teeth may remain asymptomatic or precipitate a diverse range of pathological conditions, including dental caries, pericoronitis, cysts, neoplastic lesions, and resorption of adjacent tooth roots [13,21]. When near neural structures, complications like nerve paresthesia may occur [22]. Timely detection via comprehensive clinical and radiographic assessment is therefore essential for planning an optimal management strategy, which may entail options like surgical extraction, exposure with orthodontic alignment, or autotransplantation [23,24,25]. The choice of surgical approach is guided by factors such as impaction depth, angulation, and clinician expertise, yet it is accompanied by potential postoperative sequelae, including edema, pain, bleeding, infection, nerve injury, retained root fragments, fractures, trismus, and sinus issues [15,26]. Injury to the mental or inferior alveolar nerves during surgery can lead to neurosensory deficits like anesthesia, paresthesia, dysesthesia, or hypoesthesia [15].

Surgical exposure, with or without orthodontic treatment, is recommended for impacted teeth that are tilted ≤ 45 degrees and are only moderately malpositioned [24]. Both open and closed surgical exposure techniques have excellent success rates and minimal post-operative morbidity [24,27]. An alternative treatment option with high success rates in certain cases is autotransplantation, which necessitates a skilled clinician and a favorable patient selection [25]. Nevertheless, this method is linked to an increased risk of ankylosis of the transplanted tooth, particularly in young patients, as well as external and inflammatory root resorption, pulp necrosis, and periodontal issues [28,29].

The choice of flap designs for the extraction of impacted teeth is a critical parameter, as it can minimize trauma to the surrounding tissues and preserve vital anatomical structures. Additionally, the clinician’s surgical skills and experience are essential factors in the successful removal of impacted teeth [30,31]. Despite the complexity of these procedures and the possible complications that may arise during or after the procedure, they are frequently performed.

PRF has gained popularity in oral surgery due to its cytokine and growth factor content, which enhances wound healing and reduces postoperative discomfort [32]. Studies suggest PRF accelerates healing, lessens postoperative pain and swelling, and maintains ridge dimensions after the extraction, aiding in socket and ridge preservation [33,34,35]. Additionally, emerging evidence indicates PRF and PRP may support nerve regeneration, potentially alleviating postoperative paresthesia [36,37]. Recent research suggests that the utilization of PRF in extraction sockets results in favorable outcomes, such as improved pain and edema management, reduced trismus, and a faster healing process [33]. Furthermore, there is an abundance of evidence that substantiates its function in averting bone dimensional alterations subsequent to extraction. As a result, PRF plays a substantial role in the preservation of ridges and the enhancement of sockets by restricting the resorption of both horizontal and vertical ridges [34,35]. In addition, recent research has shown that Platelet-Rich Plasma (PRP) and PRF may aid in the regeneration of peripheral nerves that have been injured. Growth factors, which promote nerve healing and tissue repair, are responsible for these promising results in alleviating postoperative paresthesia [36,38,39].

Several cases have been described in the literature in recent years concerning the management of impacted mandibular second premolars in proximity to the mental nerve, mainly using orthodontic treatment as the primary treatment approach [10,23]. However, in each one of these cases, patients were generally younger, a key factor that can ultimately determine the therapeutic plan, since orthodontic therapy presents higher success rates in adolescents than in adults [40]. Furthermore, the recent literature highlights the occurrence of neurosensory impairment after application of orthodontic forces to teeth close to the mental nerve or the inferior alveolar nerve [41]. In such cases, orthodontic treatment seems to be a risky intervention that requires clinical experience and thorough preoperative treatment planning. In conclusion, the aforementioned factors added to the prolonged total treatment time required for orthodontic therapy do not favor the selection of this alternative treatment approach, especially among adult patients [40].

Another recent report of a surgical approach to an impacted mandibular second premolar close to the mental nerve describes mild lower-lip paresthesia [42]. Despite the fact that the reported patient was younger in age, neurosensory damage is present, possibly owing either to the absence of healing and regenerative factors, similar to PRF, or to inappropriate surgical techniques.

In summary, individualized treatment planning and patient satisfaction are main priorities, especially when dealing with elaborate cases. Proper preoperative evaluation using the innovative imaging tool of CBCT provides diagnostic precision and determines the treatment approach. In the present case, surgical extraction followed evidence-based principles, while the inclusion of PRF in the extraction socket upgraded the treatment outcomes.

This manuscript presents several limitations that should be mentioned. Firstly, tooth #35 was asymptomatic on the examination and the patient had an unremarkable medical history. Even if the longest follow-up in this manuscript is considered more noteworthy compared to other case reports, it still remains a relatively short-term period. Another limitation is the lack of intraoperative figures to fully document the surgical procedure. Finally, the main limitation seems to be that this manuscript is based on a single case report; therefore, definitive conclusions cannot be drawn.

## 4. Conclusions

In summary, the surgical extraction of an impacted second premolar—often an incidental radiographic finding—necessitates prompt diagnosis, careful preoperative planning, and precise surgical technique respecting anatomical structures. Early detection broadens treatment options, including orthodontic alignment, space creation, or implant placement following surgical removal or autotransplantation. These measures are vital for preventing complications and optimizing patient outcomes. Keeping abreast of emerging research allows clinicians to refine techniques and improve success rates in managing complex cases. In our case, the impacted mandibular second premolar was surgically extracted following CBCT-based planning, ensuring a minimally invasive surgical approach. Socket management with autologous Platelet-Rich Fibrin (PRF) was associated with optimal soft tissue healing and normal neurosensory function, possibly indicating beneficial properties of PRF to accelerate and promote healing, decrease complication prevalence, and to give aid to neural regeneration.

## Data Availability

The original data presented in the study are included in the article, further inquiries can be directed to the corresponding author.

## Abbreviations

The following abbreviations are used in this manuscript:

OPG	Orthopantomogram
CBCT	Cone-Beam Computed Tomography
PRF	Platelet-Rich Fibrin
PRP	Platelet-Rich Plasma

## References

[1] Rathee M., Jain P. (2023). Embryology, Teeth. StatPearls.

[2] Huang X.-F., Chai Y. (2012). Molecular regulatory mechanism of tooth root development. Int. J. Oral Sci..

[3] Roulias P., Kalantzis N., Doukaki D., Pachiou A., Karamesinis K., Damanakis G., Gizani S., Tsolakis A.I. (2022). Teeth Eruption Disorders: A Critical Review. Children.

[4] Chen H.M., Hwang M.J., Chiang C.P. (2024). Risk factors and treatments for impacted permanent second molars. J. Dent. Sci..

[5] Möhlhenrich S.C., Korkmaz V.-C., Chhatwani S., Danesh G. (2023). General correlation between neonatal factors, primary and permanent tooth eruption and their interrelation in a population in German orthodontic practices. BMC Oral Health.

[6] Siotou K., Kouskouki M.-P., Christopoulou I., Tsolakis A.I., Tsolakis I.A. (2022). Frequency and Local Etiological Factors of Impaction of Permanent Teeth among 1400 Patients in a Greek Population. Dent. J..

[7] Alalola B.S., Almasoud F.S., Alghamdi K.B., Almalki L.M., Alodan Y.A., Alotaibi S.N., Alali S.R. (2023). Comparing the prevalence of impacted teeth through radiographic evidence among orthodontic and general populations: A secondary data analysis. Saudi Dent. J..

[8] Kaczor-Urbanowicz K., Zadurska M., Czochrowska E. (2016). Impacted Teeth: An Interdisciplinary Perspective. Adv. Clin. Exp. Med..

[9] Shekhar M.G., Pavankumar P.S., Chowdary V.J., Shivappa A.B. (2020). Bucco-lingual Impaction of Mandibular Second Premolar: Case Report and Review of Literature. Eur. J. Dent. Oral Heal..

[10] Janosy A.M., Moca A.E., Juncar R.I. (2024). Early Diagnosis and Treatment of Mandibular Second Premolar Impaction: A Case Report. Diagnostics.

[11] Chu F.C., Li T.K., Lui V.K., Newsome P.R., Chow R.L., Cheung L.K. (2003). Prevalence of impacted teeth and associated pathologies—A radiographic study of the Hong Kong Chinese population. Hong Kong Med. J. Xianggang Yi Xue Za Zhi.

[12] Vandana S., Muthu M.S., Kandaswamy D., Narayanan M.A. (2024). Proposal for a grading system to determine the clinical status and sequence of permanent teeth eruption: A pilot study. J. Oral Biol. Craniofacial Res..

[13] Santosh P. (2015). Impacted Mandibular Third Molars: Review of Literature and a Proposal of a Combined Clinical and Radiological Classification. Ann. Med. Health Sci. Res..

[14] Mahdey H.M., Wei M., Muzaffar D., Ramachandra S.S., Jameel R.A., Tanwir F., Hashmi S. (2016). Transient Paresthesia after Surgical Removal of Embedded Supernumerary Tooth. Periodontics Prosthodont..

[15] Kasapoğlu Ç., Brkić A., Gürkan-Köseoğlu B., Koçak-Berberoğlu H. (2013). Complications Following Surgery of Impacted Teeth and Their Management. A Textbook of Advanced Oral and Maxillofacial Surgery.

[16] Goswami P., Chaudhary V., Arya A., Verma R., Vijayakumar G., Bhavani M. (2024). Platelet-Rich Fibrin (PRF) and its Application in Dentistry: A Literature Review. J. Pharm. Bioallied Sci..

[17] Hajibagheri P., Basirat M., Tabari-Khomeiran Z., Asadi-Aria A. (2025). The efficacy of platelet-rich fibrin (PRF) in post-extraction hard and soft tissue healing and associated complications: A systematic review and meta-analysis of split-mouth randomized clinical trials. BMC Oral Health.

[18] Ye L., He Y., Ma W., Zhou F., Liu J. (2024). Effect of platelet-rich fibrin on the recovery after third molar surgery: A systematic review and meta-analysis. J. Cranio-Maxillofac. Surg..

[19] Yin Y., Shuai F., Liu X., Zhao Y., Han X., Zhao H. (2025). Biomaterials and therapeutic strategies designed for tooth extraction socket healing. Biomaterials.

[20] Dohan Ehrenfest D.M., Pinto N.R., Pereda A., Jiménez P., Del Corso M., Kang B.-S., Nally M., Lanata N., Wang H.-L., Quirynen M. (2018). The impact of the centrifuge characteristics and centrifugation protocols on the cells, growth factors, and fibrin architecture of a leukocyte- and platelet-rich fibrin (L-PRF) clot and membrane. Platelets.

[21] Al-Abdallah M., AlHadidi A., Hammad M., Dar-Odeh N. (2018). What factors affect the severity of permanent tooth impaction?. BMC Oral Health.

[22] Aziz S.R., Pulse C., Dourmas M.A., Roser S.M. (2002). Inferior alveolar nerve paresthesia associated with a mandibular dentigerous cyst. J. Oral Maxillofac. Surg..

[23] McNamara C., McNamara T.G. (2005). Mandibular Premolar Impaction: 2 Case Reports. J. Can. Dent. Assoc..

[24] Abu-Hussein M., Watted N., Emodi O., Awadi O. (2015). Management of Lower Second Premolar Impaction. J. Dent. Coll. Azamgarh..

[25] Bokelund M., Andreasen J.O., Christensen S.S.A., Kjær I. (2013). Autotransplantation of maxillary second premolars to mandibular recipient sites where the primary second molars were impacted, predisposes for complications. Acta Odontol. Scand..

[26] Choi J.F., Park D.Y., Williams S.L., Chang P. (2025). Oral Surgery, Extraction of Unerupted Teeth. StatPearls.

[27] Davidopoulou S. (2021). Surgical Exposure of Impacted Mandibular Second Premolar. Int. J. Clin. Stud. Med. Case Rep..

[28] Nimčenko T., Omerca G., Varinauskas V., Bramanti E., Signorino F., Cicciù M. (2013). Tooth auto-transplantation as an alternative treatment option: A literature review. Dent. Res. J..

[29] Saccomanno S., Valeri C., Di Giandomenico D., Fani E., Marzo G., Quinzi V. (2024). What is the impact of autotransplantation on the longterm stability and patient satisfaction of impacted canines? A Systematic Review. Saudi Dent. J..

[30] Ye Z.-X., Qian W.-H., Wu Y.-B., Yang C. (2021). Pathologies associated with the mandibular third molar impaction. Sci. Prog..

[31] Gojayeva G., Tekin G., Kose N.S., Dereci O., Kosar Y.C., Caliskan G. (2024). Evaluation of complications and quality of life of patient after surgical extraction of mandibular impacted third molar teeth. BMC Oral Health.

[32] Yadav R., Verma U., Dixit M., Gupta A. (2017). Platelet-rich Fibrin: A Paradigm in Periodontal Therapy—A Systematic Review. J. Int. Soc. Prev. Community Dent..

[33] Egierska D., Perszke M., Mazur M., Duś-Ilnicka I. (2023). Platelet-rich plasma and platelet-rich fibrin in oral surgery: A narrative review. Dent. Med. Probl..

[34] Al-Maawi S., Becker K., Schwarz F., Sader R., Ghanaati S. (2021). Efficacy of platelet-rich fibrin in promoting the healing of extraction sockets: A systematic review. Int. J. Implant Dent..

[35] Ucer C., Khan R.S. (2023). Extraction Socket Augmentation with Autologous Platelet-Rich Fibrin (PRF): The Rationale for Socket Augmentation. Dent. J..

[36] Logvynenko I., Bursova V. (2024). Inferior alveolar nerve injury after sagittal split osteotomy of the mandible: A literature review. Chin. J. Plast. Reconstr. Surg..

[37] Tabrizi R., Pourdanesh F., Jafari S., Behnia P. (2018). Can platelet-rich fibrin accelerate neurosensory recovery following sagittal split osteotomy? A double-blind, split-mouth, randomized clinical trial. Int. J. Oral Maxillofac. Surg..

[38] Huang Y., Bornstein M.M., Lambrichts I., Yu H.Y., Politis C., Jacobs R. (2017). Platelet-rich plasma for regeneration of neural feedback pathways around dental implants: A concise review and outlook on future possibilities. Int. J. Oral Sci..

[39] Franchini M., Cruciani M., Mengoli C., Masiello F., Marano G., D‘Aloja E., Dell’Aringa C., Pati I., Veropalumbo E., Pupella S. (2019). The use of platelet-rich plasma in oral surgery: A systematic review and meta-analysis. Blood Transfus..

[40] Becker A., Chaushu S. (2003). Success rate and duration of orthodontic treatment for adult patients with palatally impacted maxillary canines. Am. J. Orthod. Dentofac. Orthop..

[41] AlAli A.M., AlAnzi T.H. (2021). Inferior alveolar nerve damage secondary to orthodontic treatment: A systematic scoping review. Int. J. Risk Saf. Med..

[42] Rajanikanth K., Bhola N., Shukla D. (2024). Concurrent Impaction of the Mandibular Primary Second Molar and Second Premolar in Close Approximation to the Mental Nerve: A Case Report. Cureus.

